# Multispectral imaging and objective assessment of immune-tumor interactions in non-small cell lung cancer

**DOI:** 10.1186/2051-1426-3-S2-P394

**Published:** 2015-11-04

**Authors:** Carmen Ballesteros-Merino, Michael Neuberger, Zipei Feng, Rudolf Maria Huber, Julia Stump, Amanda Tufman, Rudolf Hatz, Michael Linder, Rachel Sanborn, Sanaa Hussain, John R Handy, Walter Urba, Bernard Fox, Carlo Bifulco, Simone Reu, Hauke Winter

**Affiliations:** 1 Robert W. Franz Cancer Research Center, Earle A. Chiles Research Institute, Providence Cancer Center, Portland, Oregon, USA; 2 Department of General, Visceral, Transplantation, Vascular and Thoracic Surgery, Hospital of the Ludwig Maximilian University, Munich, Germany Comprehensive Pneumology Center (CPC) and Member of the German Center for Lung Research, Munich, Germany; 3 Comprehensive Pneumology Center (CPC) and Member of the German Center for Lung Research, Munich, Germany Division of Respiratory Medicine and Thoracic Oncology, Department of Internal Medicine V, Thoracic Oncology Centre LMU Munich, Munich, Germany; 4 Comprehensive Pneumology Center (CPC) and Member of the German Center for Lung Research, Munich, Germany Asklepios Clinic Munich-Gauting, Germany, Munich, Germany; 5 Earle A. Chiles Research Institute, Portland, OR, USA; 6 Earle A. Chiles Research Institute, Providence Cancer Center, Portland, OR, USA; 7 Comprehensive Pneumology Center (CPC) and Member of the German Center for Lung Research, Munich, Germany Institute of Pathology, University of Munich, Munich, Germany, Munich, Germany

## 

Lung cancer is currently the most common cause of cancer-related death in the world and approximately 85% of all lung cancers are non-small cell lung cancer (NSCLC). Previous studies have shown that patients with NSCLC containing high densities of CD8+ memory T cells have a survival advantage. Others have identified an association between relatively high numbers of regulatory T cells and poor prognosis. Our group is interested in better characterizing the immune infiltrates and refining prognostic biomarkers that identify patients at risk of recurrence. Recent advances in multispectral imaging provide an opportunity to evaluate up to 7 markers in a single 4 micron section of formalin-fixed paraffin-embedded (FFPE) tissue. Using this technology, we reported that a high ratio of CD8:FoxP3 at the tumor correlated with the ability to isolate tumor-specific T cells from melanomas (Feng et al., submitted). This determination could be further improved by incorporating the ratio of CD8:PD-L1 present in the tumor into the evaluation. Now we are applying these same strategies to the study of NSCLC. Initial analyses have been performed on more than 450 tumor cores from 77 patients with Stage 3A/3B/4 NSCLC and included the following markers: CD3, CD8, FoxP3, CD163 and PD-L1. Additional markers are being evaluated. Ultimately, we expect that this approach could be used to stratify patients for clinical trials. We anticipate that some biomarkers will identify suppressive pathways and be associated with a short progression-free survival. While we expect there will be heterogeneity in escape mechanisms, identification of a specific mechanism could be used to tailor therapy with an agent or agents to overcome the specific immune suppressive pathway operational in that specific tumor. Patients who lack evidence for a specific suppressive pathway would be randomized to treatment with combination immunotherapy that includes a cancer vaccine.

## Author's Contribution

Drs. Ballesteros-Merino and Neuberger; and Drs. Reu and Winter contributed equally to this work. Supported by the Harder Family, Lynn and Jack Loacker, Robert W. Franz, Wes and Nancy Lematta, Providence Medical Foundation.

